# Preclinical Models for Investigation of Herbal Medicines in Liver Diseases: Update and Perspective

**DOI:** 10.1155/2016/4750163

**Published:** 2016-01-28

**Authors:** Hor-Yue Tan, Serban San-Marina, Ning Wang, Ming Hong, Sha Li, Lei Li, Fan Cheung, Xiao-Yan Wen, Yibin Feng

**Affiliations:** ^1^School of Chinese Medicine, Li Ka Shing Faculty of Medicine, The University of Hong Kong, Pokfulam, Hong Kong; ^2^Zebrafish Centre for Advanced Drug Discovery, Keenan Research Centre for Biomedical Science, Li Ka Shing Knowledge Institute, St. Michael's Hospital and Department of Medicine, University of Toronto, Toronto, ON, Canada

## Abstract

Liver disease results from a dynamic pathological process associated with cellular and genetic alterations, which may progress stepwise to liver dysfunction. Commonly, liver disease begins with hepatocyte injury, followed by persistent episodes of cellular regeneration, inflammation, and hepatocyte death that may ultimately lead to nonreversible liver failure. For centuries, herbal remedies have been used for a variety of liver diseases and recent studies have identified the active compounds that may interact with liver disease-associated targets. Further study on the herbal remedies may lead to the formulation of next generation medicines with hepatoprotective, antifibrotic, and anticancer properties. Still, the pharmacological actions of vast majority of herbal remedies remain unknown; thus, extensive preclinical studies are important. In this review, we summarize progress made over the last five years of the most commonly used preclinical models of liver diseases that are used to screen for curative herbal medicines for nonalcoholic fatty liver disease, liver fibrosis/cirrhosis, and liver. We also summarize the proposed mechanisms associated with the observed liver-protective, antifibrotic, and anticancer actions of several promising herbal medicines and discuss the challenges faced in this research field.

## 1. Introduction

Hepatic disease refers to a constellation of disorders of the liver that can lead to decompensated liver function. The liver is a very important organ that is mainly responsible for vital functions such as detoxification and glucose and lipid metabolism as well as the synthesis of many key enzymes that regulate these metabolic processes. Acute liver disease is defined as a rapid hepatic dysfunction that occurs in the absence of previous history of chronic liver disease; it is caused, for example, by excessive consumption of antibiotics or acetaminophen. By contrast, chronic liver disease is a long-term dynamic process that involves persistent hepatocytic destruction and regeneration. Major risk factors for chronic liver disease are hepatitis B viral and hepatitis C viral (HBV and HCV) infection and alcoholic liver-induced injury leading to alcoholic liver disease (ALD) as well as a constellation of metabolic disorders that can lead to nonalcoholic fatty liver disease (NAFLD). Liver exposure to these risk factors gradually results in hepatocytic injury associated with tissue infiltration of inflammatory cells and altered transcriptome in the affected cell populations. As a result, both liver scarring and regeneration are triggered which, if left unchecked, will ultimately progress to profound changes in liver architecture and liver cirrhosis. In addition, patients with cirrhosis have a higher risk of developing hepatocellular carcinoma (HCC) [1].

The incidence of NAFLD is highest among all chronic liver diseases in the United States where it was responsible for 75% of all cases in 2008 [2]. Globally, the prevalence of NAFLD ranges from 10 to 35% depending on different diagnostic tools and populations studied. For nonalcoholic induced steatohepatitis (NASH), between 3 and 5% of the global population is at risk [3]. In clinic, pioglitazone or vitamin E is only given to patients with advanced stage of NASH who failed lifestyle intervention due to the potential risk of the treatment in inducing stroke [4]. Global mortality from liver cirrhosis rose to over 1 million in 2010, accounting for 2% of all deaths worldwide. Approximately 16 out of every 100,000 people died due to liver cirrhosis worldwide, and the incidence is greater in South and Central Asia as well as Eastern European countries [5]. Liver transplantation remains the only intervention for patients with liver cirrhosis, as alternative drug therapies are not available in the clinic. Antifibrotic therapy is emerging as a possible option as several antifibrotic candidates have been shortlisted preclinically and await further study [6]. Similar to liver cirrhosis, surgical removal and liver transplantation are the most effective treatment for HCC. However, not all patients are suitable for liver surgery as the cancer may have spread and the 5-year survival rate was reported to be about 15% for patients diagnosed with HCC [7]. There is currently an unfilled medical need to find alternatives to liver transplantation by combating inflammation and the production of reactive oxygen species that are key aspects of chronic liver diseases. In many countries like China, there is a rich history of using herbal medicine to treat liver diseases. Due to the antioxidant and anti-inflammatory nature of these botanicals, their active ingredients could lead to the development of novel hepatoprotective, antifibrotic, and antiliver cancer therapies. To date, plant-based products such as Fuzheng Huayu formula and silymarin have been well documented for use in liver diseases [8]. Fuzheng Huayu formula, an FDA-approved Chinese medicinal formulation, is now undergoing phase IV clinical trial for patients with cirrhosis secondary to HBV infection in China and phase II clinical trial had been completed for chronic hepatitis C patient in US [9]. Although some herbal remedies hold promise for attenuating or reversing the progression of liver diseases, other compounds may be toxic and damage the liver [10]. Therefore, preclinical studies of dose escalation and efficacy testing should be thoroughly conducted in animal models in order to provide a better understanding of safety and efficacy before advancing herbal remedies to clinical studies.

Appropriate* in vitro* models for NAFLD and liver fibrosis remain to be developed. Furthermore, despite extensive use of immortalized cell lines and primary cultures, effective systems for high throughput drug screening are lacking. In part, this is due to the multifactorial nature of liver disease that is not amenable to investigation through simple* in vitro* cell models.

In this review, we aim to systematically review the most commonly used animal models that were employed to screen and study the efficacy of herbal medicines for liver diseases. This may serve as a guiding tool for selecting the appropriate liver disease model for herbal medicine screening as well as facilitating further exploration of studied herbal remedies for clinical applications.

## 2. Animal Models for NAFLD and NASH

NAFLD is an idiopathic pathological condition where excessive fat deposition in the liver is caused by factors other than chronic alcohol consumption. If untreated, NAFLD may further progress to nonalcoholic steatohepatitis or NASH and then liver fibrosis/cirrhosis and eventually liver cancer. NAFLD is more prevalent in patients with obesity, diabetes, insulin resistance, and hypertension, which suggests that it is the manifestation of an untreated underlying metabolic dysfunction.

Steatosis or the abnormal accumulation of fats in hepatocytes is thought to be the original disease-causing event leading to further dysfunction through oxidative stress, fatty acid and inflammatory cytokine-mediated liver injury, and apoptosis as well as changed lipid partitioning [11]. Steatosis divides into two general types: macrovesicular and microvesicular, which differ with respect to the number and size of lipid vacuoles and to the position of the nucleus in the cytoplasm. In macrovesicular steatosis, lipid vacuoles are large and the nucleus tends to push aside, while in microvesicular steatosis the nucleus is usually not affected. NAFLD accounts for approximately 5% of all liver steatosis [12]. However, in NASH, several other pathologies were observed, such as hepatocellular ballooning and intralobular and inflammatory infiltration with immune cells [13].

As mentioned earlier, liver pathology can progress from obesity and insulin resistance to macrovesicular/microvesicular steatosis, hepatocellular ballooning, and intralobular inflammation. At the biochemical level, there is a malfunction in lipid metabolism consisting of increased long chain fatty acid influx and altered fatty acid synthesis as well as decreased triglyceride production. Broadly, two types of animal models for NAFLD/NASH have been described but neither faithfully reflects the human conditions. In one type of the model, the disease is induced by a genetic modification that targets any one of the processes mentioned above while in the other it is caused by altered dietary intake. The ob/ob, db/db, adiponectin null, and KK-Ay models have been routinely employed to study the development of NAFLD. However, these models do not directly progress to steatohepatitis but require additional insults, such as various dietary treatments or the injection of hepatotoxins. On the other hand, diet-based models such as the methionine and choline deficiency (MCD) model or the high fat diet (HFD) model are easier to develop and present some of the histological features of human diseases. However, disease development in these models can vary substantially based on the composition of the diets and the duration of experiments.

The HFD model is most commonly used to explore the effect of herbal medicines on NAFLD and NASH. After 4 months, total fat/body weight was found to be five times higher in experimental HFD mice than in naïve mice. HFD mice had higher total serum cholesterol, triglyceride, and insulin levels as well as impaired glucose tolerance as compared to naïve mice. All these suggest pathological initiation of NAFLD. Administration of rhein, from* Rheum palmatum* L. after 40 days of HFD, normalized liver fat levels and improved insulin resistance [14]. In another study by Xiao et al. [15], the histopathological modifications of NASH such as hepatocytic necrosis and infiltration with inflammatory cells were observed after 8 weeks of HFD. Collagen formation detected by Sirius red staining also suggested that the disease was progressing from steatohepatitis to hepatic fibrosis. Treatment with* Lycium barbarum* polysaccharides for 4 weeks reduced insulin resistance and obesity as well as improving the histopathological changes incurred by HFD. Herbal medicines have also shown promising results in the MCD mouse model, where as previously mentioned, methionine and choline are absent from diet [16]. These components are required for beta-oxidation and VLDL synthesis and their dietary suppression leads to fatty acid accumulation and oxidative stress in hepatocytes. A recent study showed that, after eight weeks of MCD, there was observable macrovesicular steatosis, inflammation, and hepatocytic necrosis. Administration of Fuzheng Huayu formula ameliorated these changes through downregulation of CYP2E1 and HO-1, markers of oxidative stress. In addition, TNF-*α* and IL-6 were similarly downregulated in this study [17].

Animal models in which liver disease is induced by a combination of selected genetic backgrounds, diets, and the administration of hepatotoxins have also been proposed [18, 19]. In the study by Ma et al., ApoE (−/−) mice with HFD showing signs of NAFLD had ameliorated pathological changes after being fed with Huanglian Jiedu extract [20]. One possible explanation for this effect is promotion of phagocytosis by the increased population of M2 macrophages. Similarly, administration of the Japanese herbal medicines Sho-saiko-to and Juzen-taiho-to in MCD-fed db/db mice for 4 weeks reduced lobular inflammation and liver ballooning [21]. In the above models, SREBP-1c, adiponectin, PGC-1 alpha, and FABP were frequently used as biochemical markers to monitor lipid and energy metabolism functions. Overexpression of SREBP-1c induced lipid synthesis and reduced VLDL efflux and lipid oxidation while increasing triglyceride deposition in hepatocytes [22]. Adiponectin is another important cytokine that regulates glucose metabolism and fatty acid breakdown. It is reported that susceptibility for liver fibrosis in adiponectin knockout mice is higher compared to naïve mice [23]. A Japanese herbal medicine, bofutsushosan, ameliorated hepatic steatosis and inflammation through increased plasma adiponectin levels and reduced SREBP-1c expression. Simultaneously, bofutsushosan also enhanced fatty acid oxidation and reduced inflammation through upregulation of *α*/*λ*-PPAR [24]. Similar findings were reported for ping-tang recipe that greatly enhanced *α*/*λ*-PPAR expression while reducing that of the lipogenic genes, SREBP-1c, FAS, and L-FABP [25]. Proposed mechanisms for the observed effects of several herbal medicines against NAFLD and NASH together with the investigated animal models are summarized in Table 1.

## 3. Animal Models for Liver Fibrosis and Cirrhosis

Liver fibrosis is a progressive disease that is caused by viral induced hepatitis, alcoholic induced liver injury, NAFLD/NASH, chronic biliary retention, or parasitic infection induced injury [26]. The underlying pathophysiological process consists of overlapping cycles of wound healing and cell necrosis that ultimately leads to accumulation of extracellular matrix containing collagen and other matrix components [27]. An important event in fibrogenesis is the formation of hepatic myofibroblasts that secrete collagen. If left unchecked, the process results in the complete destruction of liver architecture with hepatic insufficiency that is the marker of a more severe process of cirrhosis. Ultimately, portosystemic shunting leads to liver failure [28]. To date, there is no effective treatment for liver fibrosis or cirrhosis. Whether or not these processes are reversed or arrested in their progression is a matter of current controversy. Yet, animal studies have shown that in some cases experimentally induced liver fibrosis can be reversed using botanical extracts.

Liver fibrogenesis can commonly be induced in animal settings by cholestasis or administration of hepatotoxins. In some studies, fibrosis was induced using longer-term models of alcoholic or nonalcoholic liver injury or by a combination of the two [29]. Recently, models of gene-modified mice were established by knocking down the MDR2 gene or by overexpressing TGF-*β*1 [30, 31]. The administration of hepatotoxins to induce liver injury in rodents is a model that is commonly used in herbal medicine studies. Liver injury by carbon tetrachloride (CCl_4_) administration is more frequently used than thioacetamide (TAA) or dimethyl/diethyl nitrosamine (DEN) due to short onset of disease development and the direct cytotoxic effect of CCl_4_ on hepatocytes. CCl_4_ induced early fibrosis can be detected within two weeks after the first administration, and 5 to 7 weeks are sufficient to detect all the physiological symptoms of liver fibrosis. Continuous administration of CCl_4_ will eventually lead to liver cirrhosis. The immediate cytotoxic effect of CCl_4_ on hepatocytes recruits inflammatory cells and induces secretion of proinflammatory cytokines leading to necrosis. Scarring occurs as a result of persistent cycles of cell death and is followed by hepatocytic regeneration/proliferation processes [27]. A study by Zhou et al. [32] showed that 6 weeks of intraperitoneal CCl_4_ injection 3 times per week resulted in multiple scarring/fibrotic events such as bridged vessels, fibrous septa, and even regenerative nodules. Treatment with Xuefuzhuyu starting at week 4 ameliorated the hepatic stellate cells (HSC) activation and ECM formation and reduced expression of *α*-SMA and collagen I. Another study conducted by Shen et al. [33] also showed that treatment with Diwu Yanggan in a 6-week CCl_4_ model attenuated epithelial-to-mesenchymal cellular transition, a commonly observed condition in fibrogenesis, through upregulation of E-cadherin and downregulation of vimentin. Compared to CCl_4_, disease modelling using TAA and DEN (a carcinogen) requires longer exposure time to induce HCC. In all these cases and irrespective of the causative agent, disease development is remarkably similar across the models. For example, intraperitoneal injection of TAA for 6 weeks increased fibrous septa, portal tract, and liver sinusoids as well as *α*-SMA staining of liver cells, the same as in the CCl_4_ model. In the TAA model, administration of kaerophyllin from* Bupleurum scorzonerifolium* reversed histopathological changes and further reduced the levels of proinflammatory cytokines TNF-*α*, IL-1*β*, and MCP-1 [34].

It is worth mentioning that Fuzheng Huayu formula, a traditional Chinese medicine that showed antifibrotic effects in clinical practice, also demonstrated significant* in vivo* effect in DEN and CCl_4_ models [35–38]. Currently, the Fuzheng Huayu formula is in a phase IV multicenter clinical trial for HBV induced liver cirrhosis [39]. In DMN-induced mice, administration of Fuzheng Huayu formula reduced inflammatory cell infiltration and collagen accumulation and lowered *α*-SMA expression. The antifibrotic effect of the extract is believed to downregulate TGF-*β*1 and p-Smad2/3 in injured tissue [35] as well as attenuating apoptosis via TNF*α* blockade [36]. Our research group has extensively studied the* Coptidis rhizoma* aqueous extract (CRAE), a traditional Chinese medicine used in clinical practice for liver disease. Simultaneous administration of CRAE and CCl_4_ for 8 weeks alleviated the formation of fibrous septa, pseudo lobes, and collagen deposition. The effect is comparable to that of bear bile, a medicine traditionally used for liver disease, which is also effective in CCl_4_ induced, cholestatic, and alcohol fed murine models [40]. The protective effect of* Coptidis rhizoma* extracts may be secondary to ameliorating oxidative stress and decreasing apoptosis [41]. Proposed mechanisms of action of herbal medicines in liver fibrosis/cirrhosis and the models used to test them are summarized in Table 2.

Liver fibrogenesis results from excessive deposition of extracellular matrix and is part of a wound healing process triggered by activation of hepatic stellate cells. The process is accompanied by cell necrosis, apoptosis, and inflammation [27]. TGF-*β*1 plays an important role in liver injury, by regulating the inflammation process, hepatocyte apoptosis, and the transformation of hepatic stellate cells to myofibroblasts. Transformed myofibroblasts secrete matrix metalloproteinases (MMPs) that degrade the extracellular matrix of normal cells and further promote deposition of fibrillar collagens [26]. Due to the large number of myofibroblasts and collagen deposited in fibrotic regions, the hepatic expressions of *α*-SMA and type I/II collagen also increase significantly. Therefore, these biochemical markers as well as the inflammatory factors MCP-1 and TNF*α* frequently used to evaluate the effect of herbal medicines in experimental liver fibrosis models.

Cholestatic models where bile efflux is impeded through induction of obstructive bile duct injury are also frequently used to study the effect of herbal medicines on biliary fibrosis. After surgical bile duct ligation, animals develop periportal hepatocyte necrosis, liver failure, and fibrosis within one week. Inflammatory cell infiltration, hepatocyte apoptosis, and collagen deposition are observed in 4 weeks after bile duct ligation. This murine surgical model is often used because it is fast and reproducible, even though it can result in high mortality within a few weeks, an outcome that does not mirror the slow progression of the disease in humans [29]. In animals, undergoing bile duct ligation with administration of huangqi extract restored expression of TGF-*β*1, *α*-SMA, albumin, and CK7 markers [42]. Proposed mechanisms for the actions of herbal medicines on biliary fibrosis and models used to study the effects are summarized in Table 3.

## 4. Animal Models for HCC

Exposure to a variety of agents can lead to malignant transformation of hepatocytes, a multistep process characterized by recurrent genetic modifications. HCC is the most common type of primary hepatic cancer. Its development is related to several risk factors such as HBV and HCV infection, alcoholic liver disease, and NAFLD as well as exposure to environmental toxins such as aflatoxins and diethyl nitrosamine [43]. The most effective treatment for HCC is surgical removal of the affected liver tissue followed by liver transplantation. Because HCC is most commonly diagnosed in late stages where extrahepatic metastasis is often present, early therapeutic interventions have not been explored at length.

Not surprisingly, some of these disease-causing agents mentioned above have been used to establish animal models for HCC. There are HBV/HCV transgenic mice models as well as models in which genetic alterations are induced through either knocking out of tumour suppressor genes or overexpressing c-myc or TGF-*α* protooncogenes. These models retain some features of the multistep processes of HCC development in humans and are frequently used to delineate the role of specific genes in hepatocarcinogenesis as well as studying the outcome of host-tumour interactions on disease progression [44]. Choedon et al. [45] used a* HBx15-c-myc* mouse model for HCC, in which a truncated HBx allele is overexpressed together with c-myc, to show that* Thapring*, a traditional Tibetan medicine, restores liver function after 10 months of treatment with concomitant reduction in serum of SOD and VEGF levels. The significant antitumour effect of* Thapring* is presumably linked to increased expression of p21^Waf1^ and the apoptosis [45].

Xenograft models in which tumour cell suspensions are implanted subcutaneously in mice have been extensively used to monitor tumour growth and effectiveness of new therapies. This is often the first-line model for anticancer agent screening* in vivo*. Recent study by the National Cancer Institute [46] showed up to 45% of anticancer agents confirmed to be clinically effective demonstrated cytotoxic effects in xenograft models of HCC. The H22 xenograft model that is established through injection with H22 cell lines is particularly attractive among other models, due to the relatively short induction time to develop solid tumours (approximately 2 weeks). Both subcutaneous and intraperitoneal injections can induce solid tumour formation [47] although the details for this cell line are not well described in the literature. Administration of* Eupolyphaga sinensis* inhibited H22 tumour growth by promoting secretion of TNF-*α* and IFN-*γ* as well as inducing apoptosis via increased Bax/Bcl-2 ratio and caspase-3 production [48]. Furthermore, coadministration of chemotherapeutic agents together with herbal medicines significantly increased the cytotoxic effects. Cao and colleagues [49] showed that coadministration of Fuzheng-Yiliu granules with low dose 5-fluorouracil potentiated antitumour activity and restored white blood cell count. Similarly, combination treatment of Chaiqiyigan granula with taxol had a stronger antitumour effect than that of taxol alone [50]. Using xenograft models, our research group has shown a potent antitumour effect of* Coptidis rhizoma* aqueous extract. The effect was associated with decreased levels of markers for cell proliferation and vessel density such as Ki67 and CD31 [51, 52].

Orthotopic models in which tumour cells are implanted directly into the organ of interest are considered more clinically relevant and better predictive models for drug efficacy because the emerging liver tumours better reflect the niche microenvironment. Although this model is more technically demanding, it recapitulates key events of the human disease, such as displacement of the normal cell population by tumour formation as well as the production of circulating metastatic tumour cells. Wang and colleagues [53] implanted HepG2 cells expressing red fluorescent protein into mouse livers and monitored tumour growth and metastasis by fluorescence imaging. Metastatic spread to pancreas and mediastinal lymph nodes was observed after 25 days of post-HepG2 implantation. Early treatment with* Celastrus orbiculatus* effectively blocked tumour growth. Similar to observations made in humans regarding the administration of chemotherapeutics in later stages of liver disease, the effect of* Celastrus orbiculatus* became less significant when the treatment was started after the tumours formed. In a previous study, we also implanted luciferase-tagged MHCC97L tumours into BalB/c nude mice in order to observe the effect of berberine, the major ingredient of* Coptidis rhizome*. The treatment effectively suppressed tumour growth and lung metastasis [54]. Some of the antitumour mechanisms of herbal medicines in liver cancer and the models used are summarized in Table 4.

## 5. Animal Models for Acute Liver Injury

There is a growing number of studies describing the effect of herbal medicines in acute liver failure. Acute liver failure is defined as a severe impairment of liver function within short duration and without a history of preexisting liver disease. Acute injury leading to liver failure may be less frequent in the clinic than other forms of liver failure and yet it is life-threatening [55]. In humans, drug-induced liver injury (e.g., from high doses of acetaminophen, antibiotics, or antituberculosis drugs) is the major cause of acute liver failure. Other leading causes are viral infections and accidental toxicity such as excessive alcohol consumption or mushroom poisoning as well as those caused by ischemic or metabolic disorders. Much current herbal medicine research focused on the hepatoprotective actions of herbal medicines in acute liver injury models induced by hepatotoxins, chemicals, or drugs. A list of hepatoprotective herbal medicines and related animal models is shown in Table 5. Most of these disease models are of short duration and include studies of acute biochemical parameters of liver injury such as liver enzyme levels (i.e., ALT and AST) and inflammatory markers as well as markers of organ health such as circulating albumin and total bilirubin. The histological changes for acute liver injury model are not as significant as the longer-term model. Yet, it could be identified through the induction of vacuole formation, infiltration of inflammatory cells, and hepatocytes necrosis and apoptosis. In many instances, the toxicity model is established either concurrently or following the administration of herbal medicines in order to determine an extract's hepatoprotective effect. For example, after 15 days of treatment with an aqueous licorice extract, mice were given an acute oral dose of CCl_4_ and sacrificed after 8 hours. Licorice-pretreated mice had significantly lower circulating liver enzymes and increased antioxidant enzymes in the liver, such as superoxide dismutase (SOD), catalase (CAT), glutathione peroxidase (GSH-Px), glutathione reductase (GR), and glutathione S-transferase (GST), indicating a hepatotoxic preventive effect from the licorice extract [56]. In acetaminophen-induced hepatotoxicity, coadministration of* Tournefortia sarmentosa* reduced CAT, SOD, and GPx antioxidant enzyme levels indicating less liver stress and decreased levels of inflammatory factors (TNF-*α*, IL-1*β*, and IL-6) [57]. Most hepatoprotective herbal medicines reverse liver injury through attenuation of injury-associated oxidative stress and inflammation resulting in lower levels of circulating and total bilirubin. Because at the moment liver transplantation is the only cure for acute liver failure, there is an urgent need to further explore the hepatoprotective effect of herbal medicines.

## 6. Discussion

### 6.1. Mechanisms Associated with Cytoprotective Effects of Herbal Medicines in Liver Diseases

Oxidative stress is one of the key drivers of liver disease pathogenesis. Damage by oxidative stress results when the natural balance between production and breakdown of reactive oxygen species (ROS) is disturbed. In NAFLD, beta-oxidation in mitochondria leads to disturbances in electron transport reactions and elevates ROS production [58]. ROS has been implicated in altered hepatocyte ploidy as well as the initiation of genomic changes that may further promote progression to HCC. In keeping with the reported deleterious effects of ROS on liver function, antioxidants contained in herbal remedies were shown to restore normal hepatocyte ploidy in NAFLD [59]. In the HFD induced NAFLD model, mice fed with excessive fat showed accelerated mitochondrial and peroxisomal fatty acids *β*-oxidation as well as increased microsomal fatty acid *ω*-oxidation [60]. Furthermore, ROS has also been implicated in the CCl_4_ model of liver fibrosis. CCl_3_ radicals resulting from administration of CCl_4_ interact with oxygen to produce highly reactive peroxy radicals that promote lipid peroxidation through removing hydrogen groups from unsaturated fatty acids [61]. One way that herbal medicine contributes to the amelioration of liver diseases is by improving antioxidant activity through chemical reduction of malondialdehyde (MDA) or by boosting glutathione S-transferase (GST) activity. High concentrations of the antioxidants, terpenes, and flavonoids in herbal medicines are likely responsible for the protective effect. In addition, herbal medicines increase the activity of many liver enzymes involved in ROS scavenging such as catalase, superoxide dismutase (SOD), and glutathione peroxide (GPx) as well as reducing ROS production via promoting formation of GSH, the antioxidant. Another instance of liver protection is associated with upregulation of UCP, a mitochondrial carrier [18, 62] involved in ROS metabolism.

Apart from oxidative stress, inflammation is another principal driver of liver disease. Persistent inflammation can be triggered through activation of resident macrophages and infiltration with immune cells from the blood stream following injury as well as the interaction of immune cells with surrounding liver tissues [27]. All these will lead to excessive secretion of proinflammatory cytokines TNF-*α*, IL-6, and IL-1*β* and further contribute to cell death. Furthermore, TGF-*β* secretion by activated monocytes was shown to stimulate hepatic stellate cells to increase collagen production [63]. These observations underscore the important role of inflammation-related host-cell interactions in liver disease. Many herbal medicines possess potent anti-inflammatory activities that attenuate cytokine or chemokine production. Although there are few studies reported on how herbal remedies regulate immune cells or hepatocytes to attenuate inflammation, several protein targets such as NF-*κ*B, STAT3, and AMPK have been suggested. By inhibiting these pathways, some herbal remedies effectively protect the liver against inflammation-induced damage.

### 6.2. *In Vitro* Models for Liver Diseases

To date, herbal medicine research in liver diseases, particularly NAFLD/NASH and fibrosis, rarely employed* in vitro* cell models because of their limitation in mimicking clinical pathogenesis. Although immortalized cell lines and primary cultures have been used for herbal medicine studies, interactions between different cell types and the influence of extracellular matrix as well as other aspects in the niche microenvironment, which are significant disease-contributing factors in humans, cannot be replicated in cell culture models. Using nonalcoholic fatty liver disease as an example, interactions between adipocytes and hepatocytes regulate secretion of free fatty acids that further promotes the transition of hepatocytes and surrounding nonparenchymal cells towards the disease state. Thus, many factors need to be considered regarding whether a cell model of the liver disease is to produce valuable translational outcome. Still, due to the simplicity of studying molecular mechanisms of disease and the ease of obtaining drug leads, a lot of efforts have been directed at establishing or optimizing* in vitro* cell models for high throughput drug screening. Xu et al. [64] established a TGF-*β*1 fibrogenesis two-cell based model that allows efficient quantification of the effects of antifibrotic drugs on 2D matrix accumulation and 3D nodule formation. The model has been employed in kidney fibroblasts for the screening of Chinese medicine compounds and herb extracts for inflammation independent antifibrotic activity [65]. Another model established by Chen et al. [66] is the “scar-in-a-jar” model that improves current fibroplasia models by incorporating* in situ* optical bioimaging for cell and collagen quantitation. Yet, neither of these two models has been used at length to screen for antifibrotic herbal remedies. A substantial leap in our ability to investigate the use of herbal medicines in liver diseases is expected to occur following the establishment of new models or improvement of existing ones.

### 6.3. Future Perspectives

Due to the limitation of cell models to provide translatable solutions for clinical applications, animal models remain important for translating a promising herbal compound into the clinic. The preferred protocol is to firstly establish a genetically modified or diet fed mouse model that exhibits some key aspect of the human pathogenesis. In humans, liver disease pathogenesis requires years of genetic evolution and cellular dysfunction in which processes such as cross talk between parenchymal and nonparenchymal liver cells as well as involvement of immune cells occur. Thus, the effects of herbal medicines on these cellular processes need to be delineated in order to provide a better understanding of the processes involved and to facilitate better translational context for human studies.

Single animal model and short duration studies that aim to establish the effectiveness of any herbal medicine are relatively unpersuasive. Typically, such studies use a narrow spectrum of outcomes with limited measurement criteria that may be poorly translatable to human studies. Clearly, even in complex models, the complete pathogenesis of a specific liver disease is rarely recapitulated and therefore more than one animal model is needed to validate the effectiveness of an herbal remedy. Several herbal medicines mentioned above show a profound reversal of liver disease markers and need to be studied further. For example, the potent antifibrotic effect of Yi Guan Jian extract that has been extensively studied in the CCl_4_ model is attributed to the blockade of fibrogenesis due to bone marrow derived fibrogenic cells in the liver [67–69]. However, this effect is oxidative stress-dependent and can only be partially reproduced by administration of CCl_4_ that severely damages the liver and does not reflect the conditions present in the human disease. Further studies using different rodent models should be implemented to eliminate the possibility that the therapeutic effect of this herbal remedy is due to model-specific bias.

Although preclinical studies have mostly used mouse models, alternative models based on zebrafish are now emerging. With a sequenced genome, the zebrafish has recently emerged as a robust vertebrate model for a variety of human diseases, including the liver diseases [70, 71]. Zebrafish is amenable to unparalleled ease of embryonic manipulation and adaptability for high throughput screens. Furthermore, the physiological similarities between zebrafish and the humans provide a strong rationale for direct drug discovery on zebrafish embryos with translational potential [72, 73]. Zebrafish studies further benefit from a vast array of newly developed research tools such as genome-wide ENU mutagenesis, transgenesis, and genome editing/gene knockout by ZFN, TALENs, and CRISPR. These tools are readily available to create gene-modified models of liver diseases that can be applied to large scale screens of herbal compounds/extract as well as mechanistic studies of disease progression [74].

Recently, zebrafish has been actively explored as models for NAFLD and/or steatosis using dietary addition of fructose [75] or by introducing a slc7a3a mutation to knockout genes involved in NO-AMPK-PPAR-*γ* signalling pathway [76]. ENU-mutagenized zebrafish larvae have also been used for genetic screens aimed at identifying novel genes that contribute to NAFLD/NASH phenotype [77]. Studies towards the development of zebrafish hepatic fibrosis model showed that administration of diethylnitrosamine resulted in 80% of treated zebrafish developing liver fibrosis after 6 weeks of treatment [78]. In an effort to build spontaneous hepatic cancer models in zebrafish, either constitutively or by using chemical inducers of liver-specific promoters, the Gong team has achieved targeted liver overexpression of a number of oncogenes such as Kras (V12) and Myc [79–82]. The Stainier lab has also developed a hepatocyte-specific activated *β*-catenin model in which 78% of transgenic zebrafish developed hepatocellular carcinoma by 6 months of age [83]. It is anticipated that these unique genetic zebrafish liver disease models and others will lead to further mechanistic studies as well as large-scale screens of herbal medicines in the near future.

## 7. Conclusion

Herbal medicines contain a wealth of empirical pharmacological outcomes distilled over centuries of practice but more research efforts should be tried in identifying the active medicinal ingredients. This rediscovery or modernization of traditional Chinese medicine holds great promise for new active compounds with cytoprotective, disease-arresting, and curative properties. There are still many challenges to establish relevant animal models for studying the efficacy of the promising compounds outlined in this review. The process of animal modelling is crucial to generate valuable translatable outcomes. Currently, no single model exists that completely recapitulates the entire pathological progression of NAFLD, NASH, cirrhosis, HCC, or acute liver injury. Therefore, data obtained from one model needs to be validated and studied further in other models. It is anticipated that, by employing new genome editing technologies such as CRISPR, more faithful animal models of liver disease will emerge that will push the current boundaries of herbal medicine research. While rodent models remain paramount for the study of drug efficacy, mechanism of action, and toxicity, new emerging zebrafish models assisted by a host of recent technologies hold great promises for high throughput screens of new bioactive compounds in herbal medicines.

## Figures and Tables

**Table 1:** Animal models of NAFLD and NASH used to investigate the effect of herbal medicines.

Model (ingredients)	Duration	Herbal medicine	Pathological and biochemical changes; mechanisms involved	Reference
*Diet induced models (high calories/fats diet)*				

10% lard oil and 2% cholesterol	4 wk	Si Jun Zi Tang (SJZ), Lizhong Tang (LZ), Linggui Zhugan Tang (LGZG), and Shen Zhuo Tang (SZ)	Reduced epididymal fat index and hepatic fats infiltration; reduced triglycerides and ALT levels.	[84]

Methionine and choline bitartrate tablets	4 wk	Alkaloids of *Rubus alceifolius*	Reduced hepatic lobule, serum ALT, AST, TNF*α*, and IL-6; hepatic SOD and MDA.	[16]

25% lard, 2% cholesterol 0.5% sodium cholate, and 25% Tween-80	56 d	*Schisandra chinensis* Baill	Reduced SOD, serum TC, and LDLC and increased hepatic MDA.	[85]

12% lard oil, 2% cholesterol, 0.2% propylthiouracil, and 0.5% bile salt	6 wk	*Sapindus mukorossi* Gaertn.	Increased fat deposition, fat-storing cells proliferation, collagen accumulation, macrovesicular steatosis, ballooning degeneration, and cytoplasmic vacuolation (model group). Reduced liver cell volume, hepatic lobules, and fat droplets (treatment group). Reduced TC, TG, LDL-C, ALT, and AST; increased APN level in treatment group.	[86]

10% lard, 2% cholesterol, 0.2% bile salts, 15% sucrose, 8% baking soybean powder	7 wk	TZQ formula (red peony root, mulberry leaf, lotus leaf, danshen root, and hawthorn leaf)	Increased hepatic steatosis, necrosis, and inflammatory cell infiltration (model group). Reduced TC, TG, LDL-C, HDL-C, and TC-HDL/HDL in treatment group.	[87]

88% normal chow plus 10% lard plus 2% cholesterol	4 wk + 4 wk treatment	Jiang Zhi Granule	Improved hepatic steatosis, reduced ALT and AST, and improved free fatty acid and triacylglycerol levels. Reduced LXR alpha and SREBP-1c.	[88]

MCD diet	8 wk	Fuzheng Huayu recipe	Model group showed disoriented lobule, macrosteatosis, hepatocyte necrosis, and inflammatory infiltration. Treatment group reduced ALT, AST and P450 2E1, TNF*α*, and IL-6. Increased HO-1. Inhibited alpha-SMA, TGF-*β*1, and Col I and Col III.	[17]

High fat diet^*∗*^	8 wk	Ping-tang recipe	Suppressed visceral fat accumulation, enhanced glucose metabolism, and ameliorated hepatic steatosis. Upregulated PGC-1 alpha, PPAR alpha, and gamma and reduced SREBP-1c, FAS, and FABP; activated AMPK and acetyl CoA carboxylase phosphorylation.	[25]

High fat and high fructose diet^*∗*^	9 wk	Puerariae radix, *Lycium barbarum*, *Crataegus pinnatifida*, and Polygonati rhizoma	Reduced fasting blood glucose and improved insulin resistance.	[89]

10% lard, 1.5% cholesterol, 0.2% sodium deoxycholate, 5% sugar, 0.05% prothiopyrimidine	10 wk (5 wk treatment)	Yiqi huoxue decoction and solutions of herbs	Hepatic fat deposit, macrovesicular steatosis, ballooning degeneration, and cytoplasmic vacuolation (model group); reduced lipid degeneration, fat droplets and smaller liver cells, and delineated hepatic lobules (treatment group).	[90]

9.3 g AIN-93MX, 2.6 g AIN-93VX, 0.5 g choline bitartrate, 1.1 g DL-methionine, 57.5 g lactalbumin hydrolysate, 117.5 g dextrose, 36.6 mL fish oil, and 4.5 g suspending agent K	12 wk	*Lycium barbarum* polysaccharides	Reduced lipid droplet accumulation, inflammatory cells infiltration, and hepatocyte necrosis as observed in model group. Reduced phosphorylation of TGF-*β*1 and *α*-SMA. Decreased SREBP-1c and PPAR*γ*2, but reduced ATGL and adiponectin; restored antioxidant enzymes; reduced NF-*κ*B, p-p38 MAPK, and enhanced autophagy.	[15]

High fat diet^*∗*^	12 wk	Chinese medicine recipes	Treatment group blocked fatty degeneration. Decreased expressions of JNK and p-JNK.	[91]

5.3 kcal/g (fat 59%, protein 16%, and carbohydrate 24%)	15 wk	Yin-Chen-Hao-Tang	Reduced hepatocyte foaming and ballooning. Reduced TNF-*α* and MCP-1. Promoted senescence marker protein-30 metabolism. Restored oxidative stress markers.	[92]

45 kcal% fat, 20 kcal% protein, and 35 kcal% carbohydrate	16 wk	Garcinia cambogia supplement	Increased collagen deposition in treatment group. Reduced obesity, but induced hepatic fibrosis (inflammation TNF-*α* and MCP-1 and oxidative stress SOD, GSH-Px, and TBARS increased).	[93]

Axungia porci 10%, cholesterol 1.5%, and bile salt 0.5%	16 wk	Chaihu-Shugan-San and Shen-ling-bai-zhu-San	Model group showed microvesicular and macrovesicular steatosis, lobular, and portal inflammation and hepatocyte ballooning while treatment group improved them. Decreased TNF-alpha and IL-6 in serum, inhibited TLR4, and activation of p38 MAPK.	[94]

High fat diet^*∗*^	16 wk	Soothing liver and invigorating spleen recipes	Reduced TC, LDL-C, TC, and TG in treatment group. Reduced TLR4 expression.	[95]

21.9 kJ/g, 60% fat, 20% protein, and 20% carbohydrate	20 wk	Troxerutin	Decreased epididymal adipose tissue mass, lipid accumulation, and lipid levels in treatment group. Suppressed NAD (+) depletion, increased NAMPT, and decreased PARP1. Increased SirT1 and AMPK and inhibited mTORC1 and *Lpin 1β*/*α*.	[96]

60% of kcal as fat with an energy density of 5.24 kcal/g	24 wk	Crude extract of *Lycium barbarum* polysaccharide	Lowered TC, LDL, TG, and DAG levels. Induced phosphorylation of AMPK, reduced nuclear expression of SREBP-1c, and increased UCP1 and PGC-1 alpha in adipose tissue.	[62]

60% kcal from lard/soybean 9.8 : 1	4 mo + 40 d treatment	Rhein	Steatotic and enlarged hepatocytes (model group); reduced lipid accumulation (treatment group). Reduced ALT, TC, LDL, and TG level in treatment group and improved insulin resistant. Suppressed SREBP-1c and LXR; inhibited T-BET, and enhanced GATA-3 and pSTAT3.	[14]

15% fat, 15% sucrose, and 2% cholesterol	12 wk	Salvia-Nelumbinis naturalis	Reduced macrovesicular steatosis, TG, LDL-C, and FFA levels. Increased IRS and Akt phosphorylation and decreased SOCS3.	[97]

High fat diet^*∗*^	*∗*	Cigu Xiaozhi pills	Model group showed adipose degeneration and inflammatory cell infiltration. Treatment group reduced TG, TC, ALT, AST, and MDA level while increased SOD and GPX. Reduced TNF*α*.	[98]

*Models developed by combined insults*				

ApoE (−/−) + high cholesterol diet^*∗*^	4 wk	Huanglian Jiedu decoction	Ameliorated pathological changes in fatty liver. Associated with increase of M2 macrophages populations.	[20]

MCD diet in db/db mice	4 wk	Sho-saiko-to (TJ-9), inchin-ko-to (TJ-135), juzen-taiho-to (TJ-48), and keishi-bukuryo-gan (TJ-25)	TJ-9 and TJ-48 reduced ALT. Necroinflammation, hepatic lobules, steatosis, and ballooning degeneration. Reduced TGF-*β*1, increased TNF*α*, IL-6, and PPAR-gamma, and reduced MDA.	[21]

High fat diet^*∗*^ + streptozotocin i.p.	4 wk	pomegranate flowers polyphenols	Reduced fat drops, non-HDL-C, and transaminase. Antioxidant ability enhanced and PON1 increased in liver.	[19]

High fat diet (10% lard and 2% cholesterol) + CCl_4_	8 wk	Dangyao (*Swertia pseudochinensis* Hara) and Shuifeiji (*Silybum marianum* Gaertn.)	Ameliorated hepatosteatosis lobules ballooning degeneration and inflammatory infiltration as observed in model group. Reduced ALT, AST, TAG, and MDA. Increased UCP2.	[18]

High fat diet (88% normal chow + 10% lard + 2% cholesterol) + CCl_4_	8 wk	Dangfei Liganning capsules	Improved steatosis, hepatocyte ballooning, and inflammatory infiltration as observed in model group. Improved MDA and ALT.	[99]

10% lard + 2% cholesterol + CCl_4_	8 wk	Soothing liver and invigorating spleen recipes	Reduced SREBP-1 and SCD-1.	[100]

High cholesterol^*∗*^ diet in KK-Ay mice	10 wk	Corosolic acid	Reduced blood cholesterol and liver cholesterol content. Inhibited activity of cholesterol acyltransferase.	[101]

High fat diet (640 kcal/100 g) + gold-thioglucose	12 wk	Bofutsushosan	Blocked hepatic steatosis, inflammation, hepatocyte ballooning, and Mallory-Denk bodies. Reduced ALT, AST, and TG levels. Induced adiponectin and its receptors, increased PPAR-*α* and PPAR-*γ*, and decreased SREBP-1c; reduced IR by phosphorylated Akt.	[24]

HF diet (19.6% carbohydrates, 18.2% proteins, and 62.2% lipids; total energy, 506 kcal/100) and 30% sucrose in drinks	12 wk	Goshajinkigan	Reduced AST. Increased body and adipose tissue weight and reduced elevated liver weight.	[102]

^*∗*^Content not specified in the paper.

**Table 2:** Animal models of liver fibrosis/cirrhosis used to investigate the effect of herbal medicines.

Model	Experiment durations	Herbal medicine	Pathological and biochemical changes; mechanism involved	Reference
*Hepatotoxins*				

Thioacetamide induced	6 wks	Kaerophyllin	Reduced collagen accumulation and fibrosis score. Reduced ALT, AST, and *α*-SMA. Reduced TNF*α*, IL-1*β*, MCP-1, ICAM-1; increased PPAR-*γ*.	[34]
	7 wks	Silybin	Reduced Tbil, ALT, and AST levels and necrotic zones and steatosis. Reversed loss of CYP3A and PXR; reduced *α*-SMA and improved anti-inflammatory cytokines secretion.	[103]
	8 wks	Ethanolic extract of rhizomes of *Z. officinale*		[104]
	8/12 wks	Magnesium lithospermate B	Reduced ALT, AST and *α*-SMA, TGF-*β*1, and collagen-*α*1 (I). Inhibited NF-*κ*B activation, MCP-1, and H_2_O_2_ induced ROS production.	[105]
	12 wks	Chunggan extract	Reduced inflammation, collagen deposition, and large septa formation. Reduced ALP, AST and Bil, MDA, iNOS, and TNF*α*; increased TGF-*β*.	[106]

DEN/DMN induced	3 wks	Jia-wei-xiao-yao-san	Reduced SOD, lipid peroxidation, and xanthine oxidase activity.	[107]
	4 wks	Yinchenhao decoction (YCHD group) and Yiguanjian (YGJ group)	Reduced Hyp and *α*-SMA. Reduced TNF*α*, PDGF, MDA, and GST activity; increased L-FABP and transferrin.	[108]
	4 wks	Huangqi decoction	Reduced hepatocyte apoptosis. Decreased apoptotic effectors, cleaved-caspase-3, *α*-SMA, and TGF-*β*1.	[109]
	4 wks	Yinchenhao decoction, Yinchen Wuling San, and Zhizi Baipi decoction	Reduced Hyp content and Tbil level.	[110]
	4 wks	Anluohuaxianwan	Reduced ALT, AST, and contents of HA. Increased MMP-2.	[111]
	4 wks	Huangqi decoction	*∗*	[112]
	4 wks	Fuzheng Huayu and Huangqi decoction	*∗*	[38]
	4 wks	Fuzheng Huayu recipe	Reduced Hyp content. Reduced TGF-*β*1, its receptor, and p-Smad2/3.	[35]
	4 wks	Xiaopi pill	Decreased ALT, AST, ALP and Tbil, and *α*-SMA. Reduced TGF-*β*1, TIMP-1, and HO-1.	[113]
	4 wks	Modified Sinisan	Reduced liver injury biomarkers.	[114]
	4 wks	Yi Guan Jian	Reduced fibrotic septa, degenerated hepatocytes, and collagen deposition. Reduced collagen *α*1-I, TIMP-1, and *α*-SMA.	[115]
	6 wks	*Vaccinium corymbosum* L.	Decreased liver apoptosis, necrosis, and proliferation. Reduced hepatic lipid peroxidation, protein oxidation, and nitrotyrosine levels; increased GST activity.	[116]
	6 wks	*Boswellia serrata* and *Salvia miltiorrhiza* extracts	Showed normal liver parenchymal architecture and only mild fibrosis. Reduced *α*-SMA, collagen I– collagen III, CTGF, TGF-*β*1, Smad3, and Smad7.	[117]
	15 wks	Gexiazhuyu decoction	Decreased GOT and GPT. Reduced collagen *α*-1 and *α*-SMA.	[118]
	*∗*	Corbrin Shugan capsule	*∗*	[119]

CCl_4_ induced	4 wks	Biejiayinzi, Gexiazhuyu Tang, and Fugan Wan	Gexiazhuyu Tang and Fugan Wan treatment improved inflammatory necrosis and fat degeneration. Reduced collagen accumulation.	[120]
	6 wks	Fuzhenghuayu decoction	Decreased ALT, AST, and Tbil. Reduced area ratio of liver fibrosis and *α*-SMA.	[37]
	6 wks	Xiayuxue decoction	Decreased Sirius red positive area. Reduced *α*-SMA and type I collagen.	[121]
	6 wks	Diwu Yanggan	Reduced ALT, AST, Hyp, and collagen deposition and tissue damage. Increased E-cadherin, TGF-nc; reduced vimentin, BMP-7, Hh ligand Shh, receptor Smo and Ptc, and Gli1.	[33]
	6 wks	Xuefuzhuyu decoction	Reduced ALT, AST, Tbil, and Hyp. No ECM deposition and reduced necroinflammatory foci. Decreased *α*-SMA, collagen I, CD31, VEGF, VEGFR-2, HIF-1 alpha, and ADMA; increased DDAH1.	[32]
	7 wks	Ethanol extract of Cortex Dictamni	Improved pathological grading. Reduced collagen deposition and Hyp content. Increased pY-STAT1.	[122]
	8 wks	Baicalin	Decreased Hy, steatosis, liver necrosis, and fibrotic septa formation. Reduced TGF-*β*1 and PPAR*γ*.	[123]
	8 wks	*Coptidis rhizoma* aqueous extract	Reduced Tbil and AST. Improved histological changes. Reduced SOD and Erk1/2 inhibition	[40]
	8 wks	*Cichorium intybus* L. extract	Reduced ALT, AST, Hyp, and histopathological changes. Increased GSH, SOD; reduced MDA. Reduced TGF-*β*1 and *α*-SMA.	[124]
	8 wks	Fuzhenghuayu decoction	Reduced ALT, AST, and hepatocyte apoptosis. Decreased collagen deposition and inflammatory cell infiltration. Reduced *α*-SMA and Hyp.	[37]
	8 wks	Acremoniumterricola milleretal mycelium	Decreased HA, laminin, and procollagen type III levels, Hyp. Improved pathological changes. Restored SOD and GSH-Px, inhibited lipid peroxidation. Decreased TGF-*β*, Smad2/3 phosphorylation and increased Smad7 inhibitor.	[125]
	8 wks	Rougan Huaqian granules	Decreased AST and HA. Reduced *α*-SMA, LN, Col I, Col III, Col IV, and MMP-2.	[126]
	8 wks	Fufang Biejia Ruangan pills	Decreased ALT, AST. Reduced collagen deposition and improved hepatic lesion. Reduced hyaluronic acid Col IV, type III procollagen laminin, TGF-*β*1, and Smad3.	[127]
	8 wks	Ganfukang	Decreased ALT and AST. Ameriolated ductular proliferation. Reduced *α*-SMA, MMP-2 and TIMP-1, synthesis of collagen, and activation of the Wnt/beta-catenin.	[128]
	8 wks	Huisheng oral solution	Inhibited collagen formation and improved liver function. Reduced Smad3, TGF-*β*1, *α*-SMA, and TIMP-1.	[129]
	8 wks	Methanol extracts of *Ficus carica* Linn. (Moraceae) leaves and fruits and *Morus alba* Linn. root barks	Reduced ALT, AST and ALP, and total bilirubin. Improved hepatocellular architecture. Restored antioxidant related content.	[130]
	9 wks	Dahuangzhechong pill	Decreased ALT, AST, HA, laminin, type IV collagen, and procollagen III and reverses hepatic fibrosis. Reduced *α*-SMA, serum TNF ASIL-13, p38MAPK, and Erk phosphorylation.	[131]
	9 wks	Xiayuxue decoction	Inhibited liver injury, fatty degeneration, and collagen deposition. Blocked CD31, vWF, VEGF, VEGFR2, DAF, *α*-SMA, and MMP-2 and MMP-9 activities.	[132]
	9 wks	Yiguanjian decoction	Increased Cu/Zn SOD, DJ-1, glutathione S-transferase Yb-1 subunit, and aldo-keto reductase family 7, A2.	[133]
	9 wks	Yiguanjian	Increased SOD, Prxd6, transferrin, and L-FABP; decreased MDA, HSP70, and HO-1.	[67]
	9 wks	Yiguanjian decoction	Reduced collagen deposition. Suppressed *α*-SMA, Col I, TIMP-1, TIMP-2, MMP-13, and MMP-14 and activities of MMP-2 and MMP-9.	[68]
	12 wks	Huganjiexian decoction	Inhibited collagen type I, collagen type III, TGF-*β*1, and PDGF-BB.	[134]
	12 wks	Xiaozheng Rongmu powder	Changes in ALT, AST, and Tbil are milder and with hepatocytes mitosis.	[135]
	12 wks	Shuganjianpifang	Decreased steatosis, collagen accumulation, ALT, AST, and Tbil. Increased Alb. Reduced BAX and increased Bcl-2.	[136]
	13 wks	Yiguanjian decoction	Reduced ALT, *α*-SMA, and collagen deposition. Decreased F480, Alb, EGFP, PKM2, Ki-67, and AFP.	[69]

*Others*				

Ovariectomized rats	8 wks	*Citrus unshiu* peel extract	Decreased hepatic lipid contents, AST, and ALT. Reduced hepatic lipid deposition.	[137]

Porcine serum induced	16 wks	Roots of *Paeonia lactiflora* and *Astragalus membranaceus*	Reduced liver damage and symptoms of liver fibrosis. Decreased HA, PC III, and Hyp. Restored SOD and GSH and inhibited lipid peroxidation.	[138]

Schistosomiasis induced	18 wks	Radix Astragali, *Salvia miltiorrhiza*, and *Angelica sinensis*	Some groups showed small reticulation and spot thickening in liver. Reduced ALT, albumin, and TBil.	[139]
	18 wks	Danggui Buxue decoction	Reversed fibrosis.	[140]

*Combination therapy*				

CCl_4_ + high lipid low protein diet	6 wks	Danggui Buxue decoction	Reduced hepatic fatty degeneration and collagen accumulation. Decreased ALT, AST, and TBil. Reduced MDA, TG, Hyp content, and MMP-2/9 activities; increased SOD.	[141]

CCl_4_, bile duct ligation, alcohol fed	7 wks	*Coptidis rhizoma* extract and bear bile	Reduced AST, Hyp, and Tbil. Decreased hepatic damage and fibrosis. Reduced peroxidative stress; increased SOD.	[41]

CCl_4_ + high fat emulsion	8 wks	flavonoids from Litsea coreana Levl	Reduced ALT, AST, HA, laminin, procollagen III N-terminal peptide, procollagenase IV, and Hyp. Reduced hepatocyte degeneration, inflammatory cell infiltration, and collagen deposition. Suppressed *α*-SMA, collagen I, TGF-*β*1, and its receptor; increased PPAR*γ*.	[142]

DMN and CCl_4_ induced	8 wks (DMN) + 10 wks (CCl_4_)	*Graptopetalum paraguayense*	Reduced ALT, AST, and Tbil. Suppressed collagen deposition.	[143]

^*∗*^Content not specified in the paper.

**Table 3:** Animal models of biliary fibrosis used to investigate the effect of herbal medicines.

Model	Experiment durations	Herbal medicine	Pathological and biochemical changes; mechanism involved	Reference
Bile duct ligation	3 days	Aqueous extract from the root of *Platycodon grandiflorum*	Blocked ALT and AST. Restored antioxidant enzymes. High dose showed lesser hepatocyte necrosis and inflammatory cell infiltration.	[144]
	2 weeks	Yang-Gan-wan	Reduced *α*1 (I) procollagen and *α*-SMA.	[145]
	2 weeks	*Artemisia capillaris*	Reduced cholestatic markers and Hyp. Blocked liver injury and collagen deposition. Reduced *α*-SMA, PDGF, and TGF-*β*.	[146]
	28 days	Green tea polyphenol	Reduced portosystemic shunting, fibrosis, intrahepatic angiogenesis, and mesenteric window vascular density. Decreased HIF-1*α*, VEGF, and phospho-Akt.	[147]
	4 weeks	Huangqi decoction	Reduced fibrosis degree. Increased Hyp content, CK7, and *α*-SMA.	[148]
	4 weeks	Huangqi decoction	Blocked collagen deposition. Reduced ALT, Tbil, and Hyp. Inhibited TGF-*β*1, its receptors, SMAD3, and pERK1/2.	[42]
	7 weeks	Inchinko-to	Decreased ALT and AST. Reduced TGF-*β*1 and *α*-SMA.	[149]

**Table 4:** Animal models of liver cancers used to investigate the effect of herbal medicines.

Model	Experiment durations	Herbal medicine	Mechanism involved	Reference
*Implantation (ectopic)*				

H22 cells	5 days	Fuzheng-Yiliu granule	Induced cell apoptosis. Antitumour effect after combined with 5-FU. Restored decreased level of WBC and lymphocytes.	[150]

H22 cells	5 days	Fuzheng-Yiliu granule	Induced apoptosis and CD3, CD4, and NK cells in peripheral blood. Increased serum IL-2 and TNF*α* levels.	[49]

H22 cells	5 days	Tagalsin	Upregulated wild type p53 and reduced Bcl-2.	[151]

H22 cells	10 days	Glycyrrhiza polysaccharide	Induced apoptosis via P53/PI3K/AKT pathway.	[152]

H22 cells	10 days	*Macrothelypteris viridifrons*	Inhibited expressions of VEGF and CD34.	[153, 154]

Hepa 1–6 cells	14 days	Yupingfeng powder	Reduced MDA, SOD activities in liver and lung.	[155]

H22 cells	14 days	*Eupolyphaga sinensis* Walker	Increased TNF*α* and IFN-*γ* and Bax/Bcl-2 ratio; activated caspase-3.	[48]

H22 cells	14 days	Radix Glycyrrhizae polysaccharide	Reduced Treg population and Foxp3. Increased serum Th1/Th2 cytokine.	[156]

H22 cells	15 days	Toosendanin, from *Melia toosendan* Sieb. et Zucc.	Reduced Bcl-2; increased Bax and Fas.	[157]

Hep3B cells	15 days	Andrographolide, from *Andrographis paniculata*	Reduced GSH; increased p-JNK, ASK1, MKK4, and c-Jun.	[158]

S180 and H22 ascites cells	15 days	*Dioscorea bulbifera* L. rhizome		[159]
	20 days	Jiedu Xiaozheng Yin	Increased expressions of cyclin D and cyclin E.	[49, 154]

Bel-7402 cells	25 days	Sulfated glycopeptide from *Gekko swinhonis* Guenther	Inhibited bFGF production.	[152, 160]

HepG2 cells	3 wks	pennogenyl saponins from rhizoma paridis	Decreased Bcl-2 and increased Bax, cleaved caspase-8, and cleaved caspase-3; decreased ERK1/2 and Akt phosphorylation and increased p38 and JNK phosphorylation.	[161]

SMMC-7721 cells	4 wks	Luteoloside from *Gentiana macrophylla*	Decreased ROS and NLRP3 inflammasome. Inactivated caspase-1 and IL-1*β*.	[162]

MHCC97L cells	4 wks	*Coptidis rhizoma* aqueous extract	Increased phosphorylation of eEF2.	[48, 51]

MHCC97L cells	4 wks	Huanglian Jiedu decoction	Inactivated eEF2, induced eEF2k and AMPK.	[52]

MHCC97-H cells	5 wks	Corilagin	Activated p-p53-p21(Cip1) -cdc2/cyclin B1.	[156, 163]
	*∗*	*Hedyotis diffusa* Willd.	Downregulated expressions of CDK2, cyclin E, and E2F1.	[157, 164]
	*∗*	Jiedu Xiaozheng Yin	Inhibited expressions of expression of Bmi1 and Wnt/beta-catenin.	[158, 165]
	*∗*	Triptolide from *Tripterygium wilfordii*	Increased ROS and caspase-3 activity; induced Bax and inhibit Bcl-2 after addition of 5-FU.	[166]
	*∗*	Invigorating spleen and detoxification decoction	Increased MHC I/MHC II expression.	[167]
	*∗*	*Livistona chinensis* seeds	Inhibited angiogenesis via reduction of VEGF-A and VEGFR-2 and inhibited Notch, Dll4, and Jagged1.	[168]

H22 cells	*∗*	*Pinus massoniana* bark extract	Induced apoptosis via caspase-dependent pathways.	[169]

Walker-256 cells	*∗*	Aitongxiao recipe	Reduced Bcl-2, survivin.	[170]

H22 cells	*∗*	Xiaoai Jiedu Recipe	Reduced CCL3, CXCL2.	[171]

Hep3B cells	*∗*	*Piper betle* leaf extracts	Induced MAPK-p73 pathway.	[172]

H22 cells	*∗*	Ciji Hua'ai Baosheng granule formula		[173]

H22 cells	*∗*	Oxytropis falcata		[174]

H22 cells	*∗*	Corn silk polysaccharides	Improved serum IL-2, IL-6, and TNF*α* levels.	[175]

HepG2/EGFP cells	*∗*	Chaiqiyigan granula	Increased Bax; reduced p53 and VEGF combined with taxol.	[50]

Primary hepatic carcinoma	*∗*	Bushen jianpi decoction	Reduced VEGF expression.	[176]

*Implantation (orthotopic)*				

HepG2 cells expressing red fluorescent protein	25 days	*Celastrus orbiculatus* Thunb.	Inhibited VEGF.	[53]

MHCC97H cells	35 days	Baicalein	Reduced MMP-2, MMP-9, and u-PA; increased TIMP-1 and TIMP-2 expressions through ameliorated phosphorylation of ERK.	[177]

Bel-7402 cells	10 wks	Jianpijiedu Fang	Increased PTEN, pFAK; and reduced PI3K.	[178]

MHCC97L cells	*∗*	Songyou yin	Restored E-cadherin and reduced N-cadherin.	[179]

*Transgenic mice model*				

X15-*myc* mice	12 mth	Thapring	Reduced Bcl2 and overexpression of p21^Waf1^.	[45]

*Others*				

25% CCl_4_ injection, 8% ethanol solution as drinking fluid for 4 weeks, and 20 weeks of 0.5% CCl_4_-8% ethanol solution as drinking fluid	8 wks	Xiaochaihu decoction	Reduced ONOO^−^, MDA, VMA, LPS-P, and ALP-C; increased ALP-A.	[180]

*N*-Methyl-*N*-nitrosourea	28 wks	*Aegle marmelos* leaf extract	Decreased IL-1*β*, IL-6, Bcl-2, and c-jun; increased p53 and IL-4.	[181]

^*∗*^Content not specified in the paper.

**Table 5:** Animal models of acute liver injury used to investigate the effect of herbal medicines.

Model	Experiment durations	Herbal medicine	Pathological and biochemical changes; mechanism involved	Reference
*Hepatotoxin induced*				

CCl_4_ induced	6 hrs	Tanreqing injection	Reduced serum liver markers and inflammation.	[182]
	10 hrs	*Lycium barbarum* polysaccharides	Reduced centrilobular necrosis and ALT level. Restored antioxidant enzymes, decreased NO and lipid peroxidation, attenuated hepatic inflammation, and reduced NF-*κ*B activity.	[183]
	24 hrs	Taohe Chengqi Tang	Reduced ALT and AST. Showed normal lobular pattern with mild degree of fat deposition, necrosis, and inflammatory cell infiltrations.	[184]
	2 days	Punarnavashtak kwath	Showed mild inflammation. Reduced ALT, AST, ALP, and SBRN. Increased glutathione, SOD, and catalase; decreased TBARS.	[185]
	2 days	Sharbat-e-Deenar	Restored glutathione, adenosine triphosphate, and G-6-Pase.	[186]
	2 days	Majoon-e-Dabeed-ul-ward	Decreased serum enzymes, albumin, and urea. Reduced necrosis, hepatocyte regeneration, and inflammation. Reduced TBARS; restored glutathione, adenosine triphosphatase, and G-6-Pase in liver.	[187]
	5 days	*Justicia schimperiana* (Hochst. ex Nees) and *Verbascum sinaiticum* Benth	Showed lesser injury and necrosis. Reduced ALT, AST, and ASP levels.	[188]
	5 days	Polydatin	Decreased ALT and AST. Blocked degeneration, necrosis, and inflammatory cell infiltration. Reduced MDA, hepatic TNF*α*, IL-1*β*, COX-2, iNOS, and NF-*κ*B; increased SOD, CAT, GPx, and TGF*β*.	[189]
	5 days	*Meconopsis integrifolia* (Maxim.) Franch	Reduced ALT, AST, ALP, and TB. Demonstrated *in vivo* antioxidant activities.	[190]
	7 days	Flowers of *Abelmoschus manihot* (L.) Medic	Decreased vacuole formation and hepatocellular necrosis. Reduced MDA, TNF*α*, IL-1*β*, and NO; increased GSH, SOD, GPx, CAT, and GST.	[191]
	7 days	Radix Tetrastigma	Decreased ALT and AST. Improved hepatic lesion. Reduced MDA, Bax, and caspase-3; increased SOD.	[192]
	7 days	Naringenin	Blocked necrosis and restored cellular arrangement. Decreased ALT, AST, and ALP. Reduced TNF*α*, iNOS, and COX-2; increased Nrf2 and HO-1.	[193]
	7 days	*Prosthechea michuacana* W. E. Higgins	Reduced hepatic serum markers and total protein.	[194]
	8 days	*Syzygium jambos*	Restored SGPT, SGOT, ALP, and total bilirubin.	[195]
	8 days	Heptapeptide from *Carapax trionycis*	Decreased ALT and AST. Reduced injury, necrosis, and ballooning degeneration. Showed moderate hypertrophy. Reduced MDA and increased GSH-Px.	[196]
	10 days	Ethanolic extract of *Eruca sativa* L.	Showed reduced inflammation. Reduced MDA.	[197]
	10 days	Fruit of *Lagenaria breviflora*	Decreased total bilirubin. Restored total protein, albumin, and globulin. Increased GSH and reduced MDA and H_2_O_2_.	[198]
	14 days	Dendrobium huoshanense	Reduced MDA; restored SOD, CAT, GPx, and GSH.	[199]
	15 days	Saponins in Radix Trichosanthis	Increased SOD and T-AOC; decreased MDA and LDH levels.	[200]
	15 days	Licorice aqueous extract	Reduced ALT, AST, and ALP. Increased total protein, albumin, and globulin. Improved liver SOD, CAT, GSH-Px, GR, GST activities, and GSH level; reduced MDA, liver hydroxyproline, and serum TNF*α*.	[56]
	3 wks	Ethanol extract of *Grewia tenax*	Improved hepatic necrosis. Reduced MDA.	[201]
	*∗*	*Ficus chlamydocarpa*	Decreased serum enzyme markers. Reduced GSH and liver MDA.	[202]
	*∗*	*Physalis peruviana*	Improved oxidative stress markers.	[203]
	*∗*	Shaoganduogan	Reduced transaminase; blocked degeneration and necrosis. Enhanced activity of Na+ -K+ ATPase, Ca2+ ATPase, and SOD; reduced MDA.	[193, 204]

*Chemical induced*				

D-galactosamine induced	14 days	Xiao-Chai-Hu Tang	Decreased serum IL-6 and TNF*α*, FasmRNA, FasLmRNA, and Bax protein but increased Bcl-2.	[205, 206]
	22 days	*Leucas aspera* (LA) Willd.	Showed reduced hepatocellular necrosis. Decreased ASAT, ALAT, ALP, TGL, TC, and TB. Elevated superoxide dismutase, catalase, glutathione peroxidase, and decreased lipid peroxidation levels in liver.	[206]
	22 days	*Brassica nigra* (L.) Koch	Decreased SGOT, SGPT, ALP, LDH, and *γ*-GT; increased total protein and albumin. Reduced hepatocyte degeneration.	[207]
	*∗*	Extracts from processed *Corni fructus*	Decreased ALT and AST. Improved liver damage. Increased SOD and reduced MDA.	[205, 208]

LPS induced	6 hours	Bai-Hu-Tang	No observable cellular necrosis and inflammatory cell infiltration. Prevents increase of IL10, TNF*α*.	[209]

D-galactosamine and LPS induced	1 hr	Echinacoside, from *Cistanche salsa*	Decreased ALT. Blocked hepatocyte apoptosis and improved histopathological changes. Reduced inflammatory markers.	[210]
	5 hrs	Xijiao Dihuang decoction	Reduced ALT and AST. Blocked liver injury.	[211]
	36 hrs	Sanhuangyinchi decoction	Decreased ALT, TBIL, and PT. Inhibits caspase-3 activity.	[212]
	3 d	Fuzheng Huayu recipe	Blocked hepatocyte apoptosis and reduced ALT and AST. Decreased MDA; improved SOD activity in liver. Decreased TNF*α* and TNFR1 protein expression.	[213]
	7 days	Sanhuangyinchi decoction	Reduced ALT, AST, TBIL, TP, and INR; increased FIB. Blocked the pathological changes. Increased SOD; decreased MDA and caspase-3 in liver.	[214]
	*∗*	Qingxia therapy	Decreased ALT, AST, and TBIL levels and hepatocyte necrosis and inflammatory cell infiltration. Reduced BAX, caspase-3 and increased Bcl-2.	[215]

*Drug induced*				

Paracetamol induced	24 hrs	*Andrographis paniculata* and *Swertia chirayita*	Reversed the elevated levels of GOT, GPT, ALP, and bilirubin. Restored liver architecture. Increased LPO; reduced SOD, catalase, GSH, and GPx.	[216, 217]
	7 d	*Nigella sativa* extract	Reduced liver enzymes and total bilirubin. Showed reduction of sinusoidal dilation, necrosis, and apoptosis.	[217]

Acetaminophen induced	24 hrs	*Tournefortia sarmentosa* Lam.	Reduced SGPT, SGOT, ALP, and bilirubin. Showed minimal fibrosis and periportal inflammation. Reduced inflammatory markers, MDA, and antioxidant enzyme levels.	[57, 218]
	8 days	*Capparis sepiaria* L.	Reduced SGPT, SGOT, and ALP.	[218]

Rifampicin induced in Wistar rat	15 d	*Euphorbia fusiformis* Buch.	Reduced SGOT, SGPT, GGTP, ALP, and total bilirubin. Showed mild degeneration and necrosis.	[219]

Antitubercular drug induced	45 d	*Hibiscus vitifolius* Linn.	Reduced AST, ALT, ALP, LDH, and total bilirubin and increased TC, TP, and albumin. Reduced necrosis and inflammatory cell infiltration. Increased catalase and SOD; reduced TARS.	[220]

*Others*				

Sodium arsenite induced in rats		*Ocimum basilicum*	Reduced ALT and AST.	[221]

Restrained stress induced in mouse	5 d	Myelophil, an ethanol extract of Radix Astragali and Salviae Radix	Reduced ALT and AST. Reduced ROS and lipid peroxidation; restored liver catalase, glutathione reduced, and peroxidase; normalised IL1*β*.	[222]

Dimethoate induced (insecticides) in guinea pigs	21 d	*Withania somnifera* extract	Reduced AST, ALT, and ALP. No observable pathological changes.	[223]

Pyrogallol induced	12 hrs	*Cotinus coggygria* Scop.	Reversed AST, ALT, ALP, and total bilirubin. Reduced Akt and STAT3.	[224]

Concanavalin-A	9 d	*Suaeda maritima* (L.) Dumort	Reduced serum liver markers. Restored liver architecture and showed less visible changes.	[225]

Naphthalene induced	30 d	*Coleus aromaticus* leaf extract	Showed no necrosis and infiltration of inflammatory cells. Reduced AST, ALT, ACP, and ALP.	[226]

Alpha-naphthylisothiocyanate (ANIT) induced	5 d	*Calculus bovis* Sativus	Decreased ALT, AST, ASP, and Tbil. Showed mild interlobular duct epithelial damages, lesser neutrophil cells infiltration, necrosis, and degeneration. Reduced MDA; increased hepatic SOD.	[227]
	9 d	Danning tablet	Reduced ALT, AST, ALP, T-Bil, and D-Bil. Showed mild necrotic and degenerative changes with lesser neutrophil infiltration. Reduced hepatic MPO, GST, GSH, and LPO; increased SOD, Gpx, and CAT activities.	[228]

^*∗*^Content not specified in the paper.
